# Nanopore genome sequencing of *Aeromonas salmonicida* strain AB001 from lesions of diseased *Acipenser baerii*

**DOI:** 10.3389/fgene.2026.1816465

**Published:** 2026-05-19

**Authors:** Akzhigit Mashzhan, Izat Smekenov, Serik Bakiyev, Kalamkas Utegenova, Aigerim Kuanbay, Ulykbek Kairov, Amangeldy Bissenbaev

**Affiliations:** 1 Scientific Research Institute of Biology and Biotechnology Problems, Farabi University, Almaty, Kazakhstan; 2 Almaty Branch of the National Center for Biotechnology, Central Reference Laboratory, Almaty, Kazakhstan; 3 Department of Molecular Biology and Genetics, Faculty of Biology and Biotechnology, Farabi University, Almaty, Kazakhstan; 4 Faculty of Natural Geography, Makhambet Utemisov West Kazakhstan University, Uralsk, Kazakhstan; 5 Center for Life Sciences, National Laboratory Astana, Nazarbayev University, Astana, Kazakhstan

**Keywords:** *Acipenser baerii*, *Aeromonas* salmonicida subspecies, average nucleotide identity, digital DNA–DNA hybridization, genome, taxonomy, TYGS

## 1 Introduction

*Aeromonas salmonicida* is a Gram-negative, short, rod-shaped bacterium belonging to the genus *Aeromonas* in the family Aeromonadaceae (Wang et al., 2020). It causes furunculosis, a severe form of septicaemia that affects fish in both freshwater and marine environments. It is responsible for high mortality rates and substantial economic losses in global aquaculture (Bartkova et al., 2017; Mancilla et al., 2025). The genus *Aeromonas* comprises a diverse group of opportunistic pathogens that have a broad host range and are widely distributed in the environment (Beaz-Hidalgo and Figueras, 2013; Janda and Abbott, 2010). Although it was historically considered to be a pathogen specific to salmon, significant morbidity and mortality associated with *A. salmonicida* have since been reported in numerous non-salmonid species (Fernández-Álvarez et al., 2016; Diamanka et al., 2014; Magariños et al., 2011).

Six subspecies of *A. salmonicida* are currently recognised: *A. salmonicida* subsp. *achromogenes*, *masoucida*, *pectinolytica*, *salmonicida*, *smithia* and *oncorhynch*i (recently described) (Pavan et al., 2000; Schubert, 1967; Austin et al., 1989; Ajmi et al., 2025). These taxa are supported by modern, genome-based taxonomic frameworks that refine the delineation of species and subspecies within the genus *Aeromonas* (Chun et al., 2018; Jain et al., 2018). The subspecies exhibit distinct pathogenic properties, genetic profiles and environmental adaptations, resulting in differences in growth temperature, virulence, antimicrobial resistance and host specificity (Mancilla et al., 2025; Vincent et al., 2014).

Typical cutaneous manifestations, such as furuncles, skin ulcers, and haemorrhages, are commonly observed in salmon infected with *A. salmonicida* subsp. *salmonicida*. These lesions frequently progress to septicaemia, leading to high mortality. The atypical subspecies (*achromogenes, masoucida, smithia, pectinolytica* and *oncorhynchi*) were differentiated based on biochemical characteristics, including reduced or absent pigmentation and the ability to grow at temperatures above 20 °C. Although they produce similar clinical signs, the atypical subspecies infect a broader range of hosts, including goldfish (*Carassius auratus*), carp (*Cyprinus carpio*), flounder (*Platichthys flesus*) and rainbow trout (*Oncorhynchus mykiss*) (Mancilla et al., 2025; Ajmi et al., 2025; Austin et al., 1998). Notably, *A. salmonicida* subsp. *pectinolytica* was originally isolated from polluted water rather than from diseased fish (Pavan et al., 2000).

Genomically, atypical *A. salmonicida* strains tend to be simpler, often carrying only one large plasmid. Despite this simplicity, they demonstrate high genomic plasticity, largely due to the abundance of insertion sequences in their chromosomes (Vasquez et al., 2022; Qin et al., 2025). Many fully characterised *A. salmonicida* subsp. *salmonicida* plasmids confer antimicrobial resistance, and all described plasmids in this subspecies harbour at least one tetracycline resistance gene (Dallaire-Dufresne et al., 2014; Reith et al., 2008). Other subspecies, particularly *masoucida* and *achromogenes*, have been detected in diverse environments and host species (Pfeiffer et al., 2018), yet their pathogenic mechanisms are not as well understood. Subspecies such as *pectinolytica* and *smithia* display unique metabolic adaptations; for example, *pectinolytica* exhibits strong pectinolytic activity and is likely to be saprophytic (Pavan et al., 2000). Despite these classifications, ongoing genomic and phenotypic studies continue to reveal additional diversity within *A. salmonicida*, suggesting that further subspecies or distinct evolutionary lineages may remain undiscovered.

This study analyzed samples collected from lesions of Siberian sturgeon (*Acipenser baerii*) at a fish farm in the Uralsk region of western Kazakhstan using Oxford Nanopore Technologies (ONT) whole-genome sequencing. The isolate obtained, designated AB001, was derived from these lesions, but no direct causal relationship between the isolate and the observed disease is inferred. Strain AB001 is an independent isolate with a distinct genome dataset that is different from that of previously reported *Aeromonas* strains associated with sturgeon, including *Aeromonas oralensis* AB005^T^ (Mashzhan et al., 2025). This genome sequence is a valuable resource for comparative analysis of *A. salmonicida* subspecies, offering new insights into their pathogenicity and evolutionary differentiation.

## 2 Materials and methods

### 2.1 Sampling

Tissue samples were collected from lesions of *A. baerii* (*A. baerii*) at a fish farm in Uralsk, in the West Kazakhstan region (45◦52′N, 51◦15′E) in June 2022, as previously described (Bakiye et al., 2023). The bacterial strain AB001 was previously isolated from these samples using Luria–Bertani (LB) agar and characterised phenotypically. The same isolate was used for whole-genome sequencing and genomic analysis in the present study.

Swabs were collected from the full depth of the external lesions and streaked onto Luria–Bertani (LB) medium. The plates were then incubated at 30 °C for 24 h. The dominant colonies obtained after three successive streakings onto LB agar plates were repeatedly subcultured to obtain a pure culture. The AB001 culture was preserved at −80 °C in LB medium supplemented with 20% glycerol.

### 2.2 DNA extraction, sequencing, and genome assembly

Genomic DNA was extracted using a Genomic DNA Extraction Kit (TransGen, China). The quality and concentration of the DNA were assessed using a NanoDrop 2000C spectrophotometer (Thermo Fisher Scientific, USA). The integrity of the DNA and the absence of RNA were verified by electrophoresis on a 1.0% agarose gel (Sigma, USA).

Whole-genome sequencing of strain AB001 was performed on the PromethION 48 platform (Oxford Nanopore Technologies, United Kingdom) at the Center for Life Sciences, Nazarbayev University, Kazakhstan, using FLO-PRO002 flow cells and the SQK-LSK109 library preparation kit as described previously (Mashzhan et al., 2025). Strain AB001 is an independent isolate obtained in the present study and is distinct from *Aeromonas* strains previously reported to be associated with sturgeon, including *A. oralensis* AB005^T^ (Mashzhan et al., 2025). The genome analyzed here is newly generated and does not constitute a reanalysis or extension of the dataset reported in (Mashzhan et al., 2025).

Raw FAST5 signal data were basecalled using Guppy Basecaller v6.2.7 with the high-accuracy model, applying a minimum quality threshold of Q ≥ 9 (Wick et al., 2019). Adapter and barcode sequences were removed using Porechop v0.2.4, followed by length filtering to exclude reads shorter than 1 kb.

*De novo* genome assembly was performed using Flye v2.9.3 (Kolmogorov et al., 2019). The resulting assembly was polished using two rounds of Racon v1.5 with filtered Nanopore reads (Vaser et al., 2017). This was followed by final consensus correction with Medaka v1.8 (high-accuracy model). This polishing strategy was employed to enhance the base-level accuracy of the long-read assembly. Circularization analysis was conducted using Circlator v1.5 (Hunt et al., 2015). Genome quality was assessed using CheckM v1.2 (Parks et al., 2015).

### 2.3 Genome annotation and comparative genomic analyses

The RASTtk pipeline was used for genome annotation, predicting protein-coding sequences and RNA genes and assigning functional annotations based on homology (Overbeek et al., 2014).

Comparative genomic analyses were performed using the publicly available genomes of representative strains of *A. salmonicida*, including type strains and reference genomes, as well as phylogenetically related subspecies. Genome selection was based on taxonomic relevance and the representation of both typical and atypical strains within the species complex. Average nucleotide identity (ANI) was calculated using FastANI (v1.33) with the default parameters (Jain et al., 2018). Digital DNA–DNA hybridisation (dDDH) values were obtained using the Type (Strain) Genome Server (TYGS) based on the Genome BLAST Distance Phylogeny (GBDP) method with the default settings (accessed 6 October 2025) (Meier-Kolthoff and Goker, 2019). Orthologous clustering analysis was conducted using OrthoVenn3, setting the E-value cutoff to 1e−5 and the inflation value to 1.5 (Sun et al., 2023). This analysis was based on predicted protein sequences derived from genome annotations. OrthoVenn3 was employed as an exploratory tool to evaluate the shared and unique gene content of the analysed genomes (accessed 20 November 2025).

### 2.4 Genome feature analysis and functional prediction

Genome feature analyses were conducted to identify mobile genetic elements and structural genomic features. Prophage regions were predicted using PHASTER (Arndt et al., 2016). CRISPR arrays were identified using CRISPRCasFinder (Couvin et al., 2018). Genomic islands were detected using IslandViewer (accessed 29 October 2025) (Bertelli et al., 2017). All analyses were performed using the default parameters. The pathogenic potential was predicted computationally using PathogenFinder2 (v2-0.5.0) (Barbour et al., 2013). Antimicrobial resistance genes were identified using ResFinder (v4.7.2) (Bortolaia et al., 2020) and the Comprehensive Antibiotic Resistance Database (CARD, v6.0.5) with the recommended identity thresholds and default parameters applied (Alcock et al., 2023).

## 3 Sequencing and genome assembly

Basecalling produced 13.06 Gb of passed reads (Q ≥ 9) and 23.64 Gb of failed reads (Q < 9) from a total of 2.9 million reads, with an estimated read N50 value of 23.31 kb. Analysis of the read length distribution showed that the longest 1% of reads ranged from 64 to 170 kb, with the majority of long reads falling within the 64–96 kb range (121.31 Mb). Smaller fractions were found in the 96–128 kb range (7.85 Mb), the 128–160 kb range (1.13 Mb) and the 160–170 kb range (0.34 Mb). The filtered dataset (∼14.8 Gb, ∼3.2 million reads) provided effective genome coverage of ∼1021×.

The genome was assembled into a single contig comprising 4,742,014 base pairs (bp), with a GC content of 58.6%. Analysis of circularisation confirmed that the chromosome is circular and that no plasmids were present. These metrics suggest that the genome assembly is of a high quality and complete. Genome quality assessment using CheckM revealed a completeness score of 96.74% and a contamination score of 0.18%, which further supports the high quality of the assembly.

### 3.1 Genome features and annotation

The RASTtk pipeline identified 4,256 protein-coding sequences, 345 of which were assigned to subsystems and 127 of which were tRNA genes (Table 1; Supplementary Figure S1). Functional categorisation revealed that many genes are associated with fundamental metabolic processes, such as amino acid metabolism, carbohydrate utilisation, protein metabolism, the biosynthesis of cofactors and secondary metabolites, and motility and chemotaxis. Genes involved in stress response mechanisms (oxidative, osmotic, detoxification and periplasmic) were also identified.

**TABLE 1:** Genome statistics of closest *Aeromonas* genomes*.

Scientific name	*Aeromonas salmonicida* AB001	*A. salmonicida* subsp. *salmonicida* CIP 103209^T^	*A. salmonicida* subsp. *smithia* ^T^	*A. salmonicida* subsp. *pectinolytica* 34mel^T^	*A. salmonicida* subsp*. masoucida* NBRC 13784^T^	*A. salmonicida* subsp. *achromogenes* JCM 7875^T^	*A. salmonicida* subsp. *oncorhynchi* strain A-9^T^
Genome size (Mb)	4.7	4.7	4.7	5	4.5	4.5	5.1
G + C content (%)	58.6	58.5	58.5	58.5	59	59	58.1
N50	4.7 Mb	4.7 Mb	119.1 kb	5 Mb	43.9 kb	44.3 kb	5.1 Mb
L50	1	1	13	1	33	34	1
Number of contigs	1	5	140	1	227	243	1
Number of coding sequences (CDS)	4256	4259	4275	4354	3953	3942	4628
Number of predicted proteins	4565	4250	4275	4254	4249	4304	4699
Singleton proteins	473	180	230	330	158	297	365
Number of tRNAs	127	116	124	126	83	83	128
5S rRNA	11	10	11	11	2	2	11
16S rRNA	10	9	10	10	1	1	10
23S rRNA	10	9	10	10	1	1	10
NCBI accession no.	JBUAPV000000000	GCA_000820065.1	GCA_048259455.1	CP022426.1	GCA_000647955.1	GCA_039523715.1	CP178319.1

* The data were retrieved from GenBank (Benson et al., 2018), RASTtk, pipeline (Overbeek et al., 2014) and orthologous clustering was performed using OrthoVenn3 (predicted proteins and singleton counts).

T denotes the type strain.

Analysis of genome features identified one intact prophage region (36.5 kb), nine CRISPR elements and 36 putative genomic islands, ranging in size from 4,170 to 18,741 bp. The largest genomic island contained 22 genes, most of which were annotated as hypothetical proteins.

A computational prediction was made using PathogenFinder2, yielding a mean probability score of 0.9379 (SD = 0.018) for human pathogenic potential based on genomic features. Screening with ResFinder, using a minimum identity threshold of 98%, identified a metallo-*β*-lactamase gene (*cphA5*) associated with carbapenem resistance. Further analysis against the Comprehensive Antibiotic Resistance Database (CARD) detected additional predicted resistance determinants, including OXA- and FOX-type *β*-lactamases, polymyxin resistance-associated genes (*arnT*), RND efflux system components (adeF-like variants) and EF-Tu variants associated with elfamycin resistance. These findings are based on *in silico* predictions derived from genome sequence data and should not be interpreted as evidence of phenotypic antimicrobial resistance or host-specific pathogenicity without experimental validation.

### 3.2 Comparative genomic context

**FIGURE 1:**
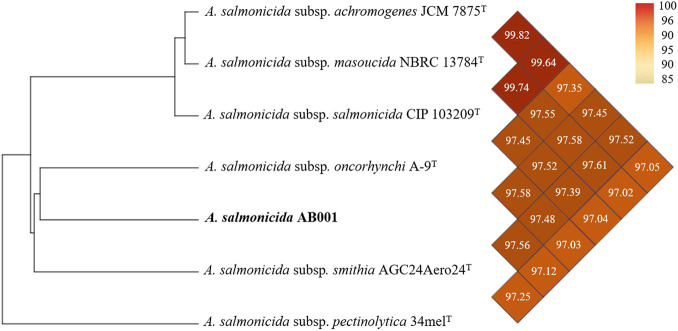
The heat map was generated using the OrthoANI values calculated by the OAT software for *Aeromonas salmonicida* strain AB001 and other closely related *Aeromonas* subspecies. The colour codes represent the OrthoANI values as a percentage.

Comparative analyses were performed using representative genomes of the *A. salmonicida* subspecies (Figure 1). Average nucleotide identity (ANI) analysis revealed that strain AB001 exhibits the greatest similarity (97.58%) to *A. salmonicida* subsp. *oncorhynchi* A-9^T^, with ANI values relative to non-*salmonicida* taxa falling below 95%.

Digital DNA–DNA hybridisation (dDDH) analysis yielded a value of 79.2% for strain A-9^T^. Together, these metrics support the assignment of strain AB001 to the *A. salmonicida* species complex, while also indicating genomic variation within the group (Supplementary Figure S2).

An orthologous clustering analysis using OrthoVenn3 identified a conserved core comprising 3,158 orthologous clusters that were shared by all seven of the analysed genomes (Supplementary Figure S3). Additionally, strain AB001 exhibited 473 strain-specific singleton protein clusters. These are defined as putative genes lacking detectable homologues in the compared genomes. This indicates potential accessory genome content and strain-specific functions.

Collectively, these observations indicate that AB001 is closely related to *A. salmonicida* subsp. *oncorhynchi*, but also exhibits distinct genomic features within the species complex.

## 4 Data value and reuse potential

This Data Report presents a high-quality, long-read assembly of the *A. salmonicida* strain AB001 isolated from diseased aquaculture fish in western Kazakhstan. The dataset includes a contiguous genome assembly with functional annotation and associated comparative genomic metrics, including ANI, dDDH, orthologous clustering and mobile genetic element prediction.

Collectively, comparative analyses indicate that strain AB001 is closely related to *A. salmonicida* subsp. *oncorhynchi*, while exhibiting measurable genomic divergence. While ANI values support its placement within the *A. salmonicida* species complex, dDDH estimates reveal variation in strain-specific gene content and the accessory genome, approaching the subspecies delineation threshold. Together with orthologous clustering results, these findings provide a coherent genomic context for AB001 as part of the intraspecies diversity of *A. salmonicida*. These additional observations provide a coherent genomic context for AB001 within the intraspecies diversity of *A. salmonicida*.

The publicly available genome sequence may be reused for comparative genomics, taxonomic benchmarking, antimicrobial resistance surveillance, and studies of genome plasticity within aquatic bacterial pathogens. Future updates or extended analyses may be deposited as independent dataset versions or reported in separate research articles.

## Data Availability

The datasets presented in this study are publicly available. This data can be found in the GenBank repository at https://www.ncbi.nlm.nih.gov/genbank/ with the accession number JBUAPV000000000.
