# Supplementary material for: Nanopore genome sequencing of Aeromonas salmonicida strain AB001 from lesions of diseased Acipenser baerii
Source: Front Genet. 2026 May 19;17:1816465. doi: 10.3389/fgene.2026.1816465 (PMC13225778; doi:10.3389/fgene.2026.1816465)
Supplement: Supplementary file 1 [file DataSheet1.doc]

Supplementary Material

# Supplementary Figures


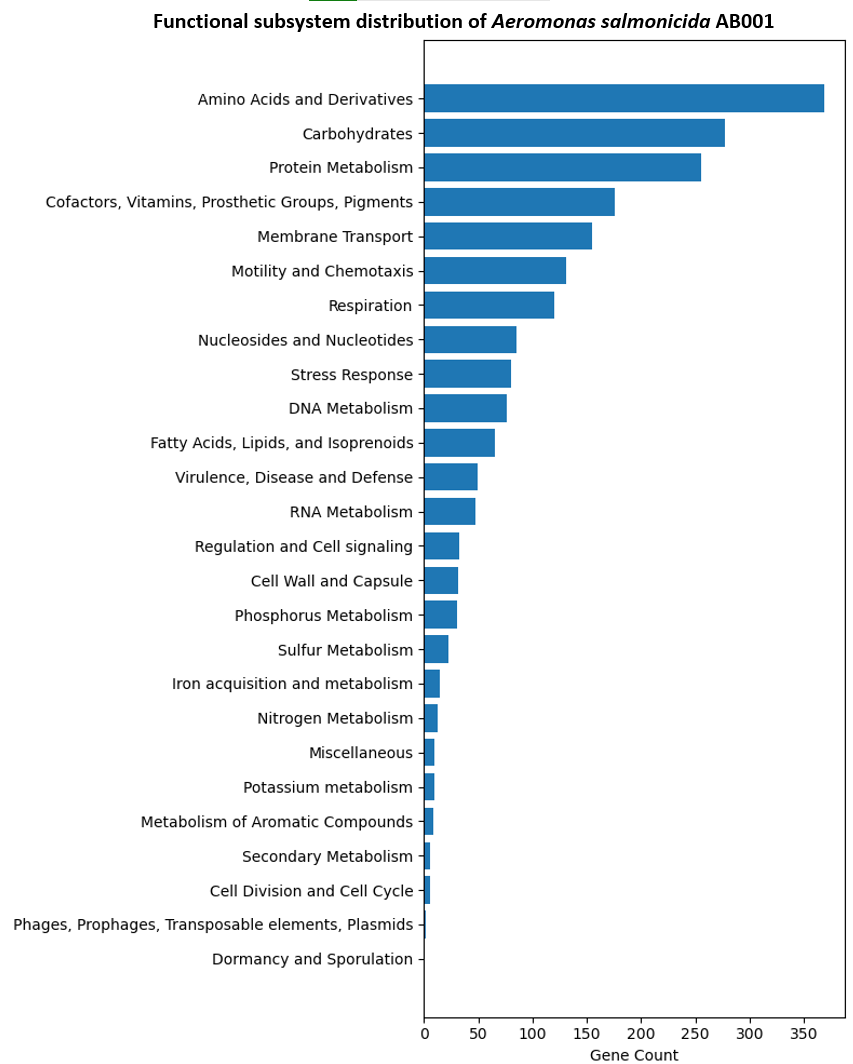


**Supplementary Figure 1.** Functional subsystem distribution of the *Aeromonas salmonicida* strain AB001 genome based on RAST annotation. The majority of the annotated genes are associated with amino acid metabolism, carbohydrate utilisation and protein metabolism, followed by cofactors and membrane transport systems. Categories with ≤10 genes are included to provide a comprehensive overview of the functional composition of the genome.


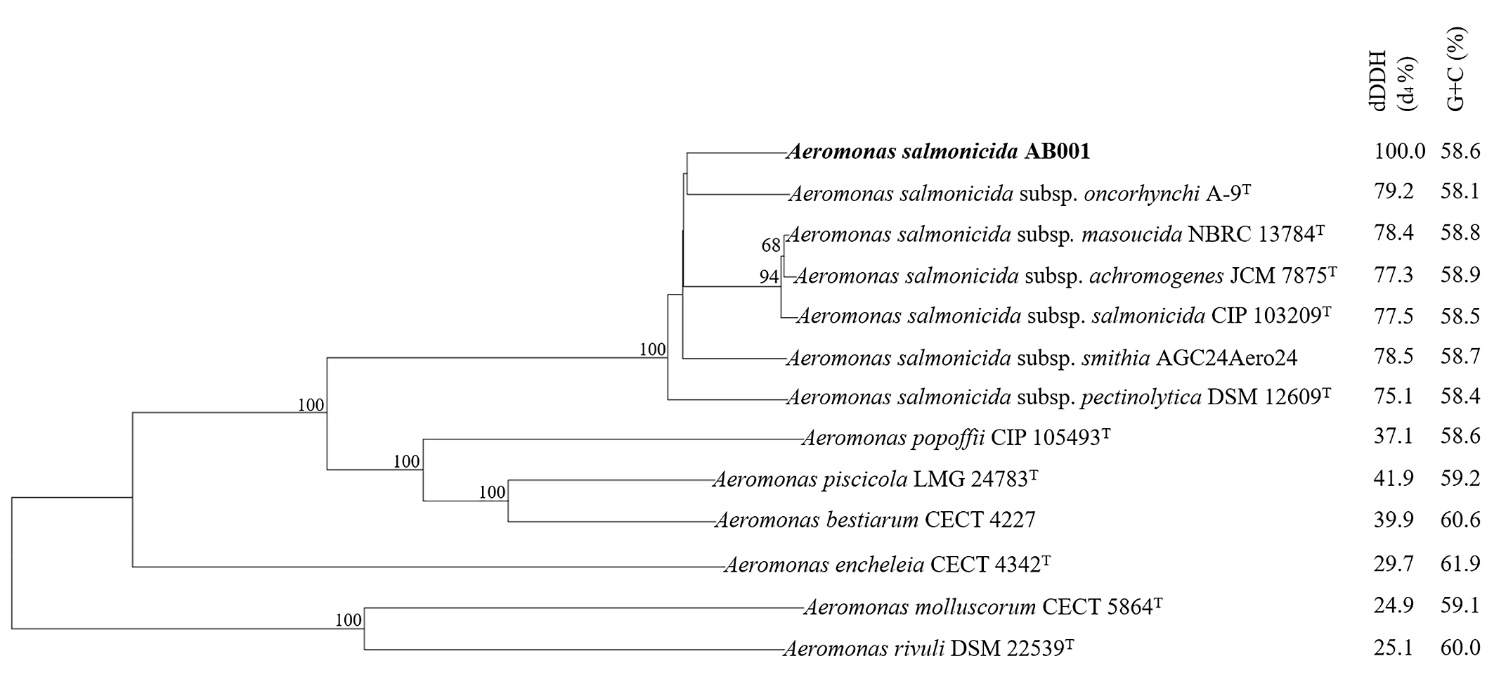


**Supplementary Figure 2.** Phylogenomic tree of *Aeromonas* *salmonicida* strain AB001 and representative members of the genus *Aeromonas*. The tree was inferred using FastME 2.1.6.1 (1) from the Genome Blast Distance Phylogeny (GBDP). The branch lengths are scaled according to the GBDP distance formula d5. The numbers above branches represent GBDP pseudo-bootstrap support values greater than 80% from 100 replications, with an average branch support of 90.14%. The tree was rooted at the midpoint (2) and visualized with PhyD3 (3).


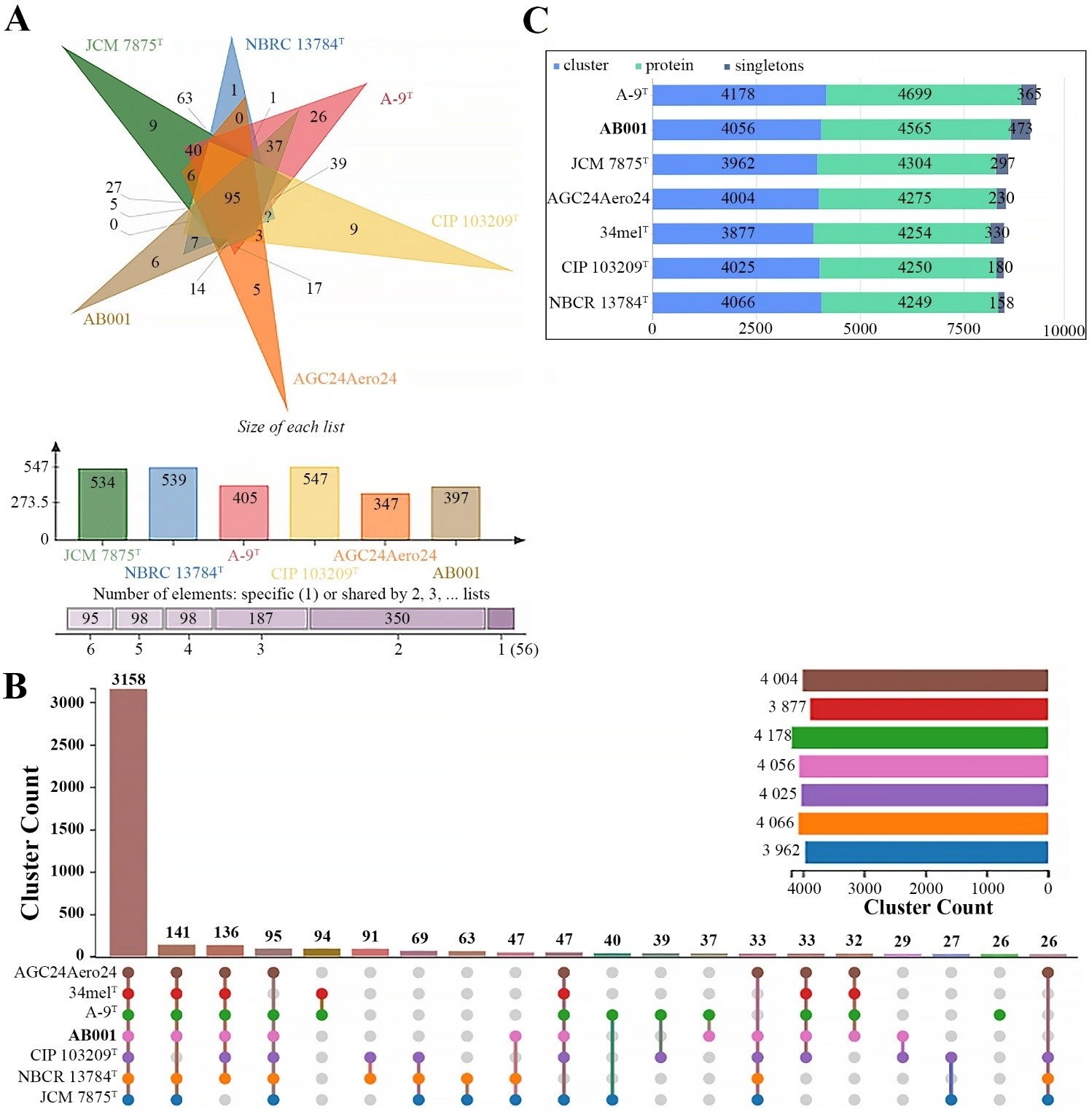


**Supplementary Figure 3.** Orthologous gene clustering analysis between the *Aeromonas salmonicida* strain AB001 and the following strains of *A. salmonicida*: *A. salmonicida* subsp*. oncorhynchi* A-9^T^, *A. salmonicida* subsp. *smithia* AGC24Aero24, *A. salmonicida* subsp. *salmonicida* CIP 103209^T^, *A. salmonicida* subsp. *achromogenes* JCM 7875^T^, *A. salmonicida* subsp. *masoucida* NBRC 13784^T^, *A. salmonicida* subsp. *pectinolytica* 34mel^T^: (A) Classic Venn diagram illustrating the selected species (B) UpSet plot displaying the unique and shared orthologous clusters among the species (C) Bar chart representing the number of protein sequences, orthologous clusters, and singletons for each species.

# References

1. Lefort V, Desper R, Gascuel O. FastME 2.0: A comprehensive, accurate, and fast distance-based phylogeny inference program. **Mol Biol Evol. 2015 Jun 30;32(10):2798–2800**. doi: [10.1093/molbev/msv150](https://doi.org/10.1093/molbev/msv150)

2. Farris JS. Estimating phylogenetic trees from distance matrices. **Am Nat.** 1972 Sep;106:645–668. doi: [10.1086/282802](https://doi.org/10.1086/282802)

3. Kreft L, Botzki A, Coppens F, Vandepoele K, Van Bel M, Kelso J. PhyD3: a phylogenetic tree viewer with extended phyloXML support. **Bioinformatics. 2017 Sep 15;33(18):2946-2947**. doi: [10.1093/bioinformatics/btx324](https://doi.org/10.1093/bioinformatics/btx324)
